# Choices Regarding Thrombolysis Are Modified by the Way to Transfer the Messages

**DOI:** 10.3389/fneur.2017.00589

**Published:** 2017-11-07

**Authors:** Jingjing Gong, Yan Zhang, Hongyan Gao, Wei Wei, Jing Lv, Hongyun Liu, Yonghua Huang

**Affiliations:** ^1^Department of Neurology, PLA Army General Hospital, Beijing, China; ^2^Center of Psychology, Air Force Aviation Medicine Research Institute, Beijing, China; ^3^Department of Medical Administration, PLA Army General Hospital, Beijing, China; ^4^Department of Psychology, PLA General Hospital, Beijing, China; ^5^School of Psychology, Beijing Normal University, Beijing, China

**Keywords:** stroke, medical decision-making, risk communication, psychosocial intervention, statistical modeling

## Abstract

Although thrombolysis is the most effective medical treatment for acute ischemic stroke, many stroke patients eligible for thrombolysis miss this treatment as a result of delay or refusal by the patients and/or their proxies. To explore the influences of prognostic information for different intervals from stroke onset to the start of thrombolytic treatment (OTT) and other factors on the preferences of patients/proxies regarding thrombolytic therapy, a cross-sectional, discrete-choice experiment was performed between August 2013 and September 2014. A total of 613 Chinese inpatients or their immediate family members were consecutively recruited at the Department of Neurology. After random assignment to a negative-framing group or a positive-framing group, the subjects completed a series of surveys, including nine items about thrombolysis. Latent class analysis (LCA) was used to examine participants’ preference paradigms for thrombolysis and to categorize the participants into different subgroups. Subsequently, regression analyses were conducted to explore predictors of categorization of the participants into each subgroup and to construct a thrombolytic decision-making model. LCA revealed an optimal 3-subgroup model including a consent to thrombolysis subgroup and objection to thrombolysis subgroups 1 and 2. Multiple regression analysis demonstrated that compared with assignment to the consent to thrombolysis subgroup, assignment to objection to thrombolysis subgroup 1 or 2 could be predicted by different factors. χ^2^ tests indicated effects of framing and other factors on participants’ choices regarding thrombolysis. Choices regarding thrombolysis were modified by not only prognostic information for different OTT intervals but also message framing, presentation format, and sociodemographic characteristics. To facilitate consent to thrombolysis, physicians should convey prognostic information to patients/proxies on the basis of patient OTT interval and should order the presentation of therapies according to the classification of patients/proxies. Individualized decision-making (IDM) might be an optimal strategy to increase the selection of thrombolysis, which providing important reference points for IDM in other clinical domains.

## Introduction

China has made great improvements in public health in the past few decades. Unfortunately, stroke, as a global disaster and the second leading cause of death after cancer worldwide (1), remains a leading cause of death in China as of 2010 (1.7 million deaths, 95% UI 1.5–1.8 million), though there have been rapid demographic and epidemiological changes in China (2). In 1996, the US Food and Drug Administration approved thrombolytic therapy using intravenous recombinant tissue-type plasminogen activator as the most effective medical treatment for acute ischemic stroke (AIS) (3). Pooled data from large randomized controlled trials and observational studies have strongly suggested that such therapy significantly increases the overall odds of a good stroke outcome when delivered within 4.5 h of stroke onset with acceptable safety (4–7). Any strategy that increases thrombolysis rates will increase both cost-effectiveness and patient quality of life (4, 8). However, there has been some reluctance to use thrombolysis more than 4.5 h after stroke onset or due to concerns over fatal intracranial hemorrhage (4). There is a low rate of thrombolysis in the US (2.4–5.2%) (9) and an even lower rate in China (1.6% or less), with longer onset-to-needle time and longer door-to-needle time in China (10). The most common reason for such delays in China is the time required to obtain consent (43.24%) (10), and 18.2% of stroke patients eligible for thrombolysis miss treatment due to delay or refusal by the patients or their proxies (11).

There is a consensus that the framing of therapeutic outcomes, namely, the presentation format of clinical trial results, might influence preferences for treatment during medical decision-making (12, 13). For example, a study examining the effects of information presentation (framing) on teratogenic risk perception in pregnant women showed that women receiving negatively framed information (i.e., 1–3% chance of having a malformed child) had a significantly higher perception of teratogenic risk than those receiving positively framed information (97–99% chance of having a normal child) and were less likely to want to take the associated drug (13). The framing effect, first experimentally confirmed in 1981 (14), can influence medical treatment options, choices of prevention and screening, and other health behaviors (15), and it varies with other internal/external factors, such as the type of scenario and patient characteristics (e.g., numeracy, emotion, social cognition, age, and education) (16, 17). Our previous study indicated that the preferences of patients or their proxies for thrombolysis is significantly influenced by the valence (positive vs. negative) of the framing scenario (thrombolysis labeled by rates of survival, no disability, and no parenchymal hemorrhage vs. by rates of mortality, disability, and parenchymal hemorrhage), the format of information presentation, and participant characteristics (18). Patients with different stroke onset to start of treatment (OTT) intervals in thrombolysis obtain different stroke outcomes (7), and earlier thrombolytic treatment results in larger proportional benefits (4, 8). However, whether differences in prognostic information within different OTT intervals influence patient preferences for thrombolytic therapy has rarely been studied and reported. This study determines whether thrombolytic decision-making is influenced by differences in prognostic information within different OTT intervals. We also examine whether prognostic information, message framing, presentation format, and sociodemographic characteristics have interactive effects on the perceptual judgment of thrombolytic therapy.

## Materials and Methods

### Participants

A total of 613 Chinese inpatients in the Department of Neurology or their immediate family members were consecutively recruited between August 9, 2013 and September 16, 2014. The general eligibility criteria for the subjects included (1) age ≥18 years; (2) normal cognition, as indicated by a Mini-Mental State Examination (Chinese revised version) score of either >20 (for those with ≤6 years of education) or >24 (for those with >6 years of education); (3) the ability to communicate verbally and complete the questionnaires; (4) no history of severe mental disorders; (5) no disability (modified Rankin Scale score of 0–1); (6) no history of thrombolytic therapy; and (7) diagnosis *via* a brain MRI scan (chronic symptomatic, lacunar, or acute cerebral infarction) if categorized into the group of stroke inpatients. Among the 613 participants, 46 were excluded (2 due to loss of the questionnaires, 3 due to age <18 years, 7 due to a history of cerebral hemorrhage, 9 due to medical work experience, and 25 due to failing to complete the assessments) (18). The PLA Army General Hospital ethics committee approved the study protocol. Each participant provided informed consent for participation in the experiment. Detailed demographic information is presented in Appendix Table I in Supplementary Material.

### Materials

The assessments included instructions; a numeracy scale (19); nine items consisting of different presentation formats of information about thrombolysis in different OTT intervals; sociodemographic, health and attitude questionnaires; and the SCL-90-R (20) (Appendix Materials in Supplementary Material; Table 1).

**Table 1:** Formats of presentation of information related to thrombolytic therapy by different OTT intervals.

Presentation format of pros and cons	Item no.	Hospital no.	OTT interval (min)	First presentation of therapy in the items	Description of parenchymal hemorrhage in pros and cons
**Percentage**					
	1	1001	91–180	Non-thrombolytic therapy	No
	2	1002	181–270	Non-thrombolytic therapy	No
	3	1004	0–90	Non-thrombolytic therapy	Yes
	4	1005	91–180	Non-thrombolytic therapy	Yes
	5	1006	181–270	Non-thrombolytic therapy	Yes

**Numeric OR**					
	6	4001	91–180	Thrombolytic therapy	Yes
	7	4002	91–180	Non-thrombolytic therapy	Yes
	8	4003	181–270	Thrombolytic therapy	Yes
	9	4004	181–270	Non-thrombolytic therapy	Yes

*OTT, stroke onset to start of treatment; OR, odds ratio*.

### Design and Procedures

The participants were randomly assigned to a negative-framing group or a positive-framing group (18), in which the participants were presented with negative or positive information about thrombolysis, respectively. They then completed a series of surveys including nine items about thrombolysis (each item contained information on the benefits and risks of thrombolysis). Regardless of the framing scenario, all participant responses to the nine items were analyzed by latent class analysis (LCA). LCA is similar to cluster analytic methods and can be used to identify different subgroups of participants (latent classes) according to their item response patterns. Those participants categorized into the homogeneous subgroup reported similar attitudes toward thrombolysis. Another goal of using LCA was to identify the optimal model, i.e., the model that contained the smallest number of subgroups necessary to adequately describe the association of the choice of thrombolysis with different OTT intervals (e.g., 91–180 and 181–270 min) and the format of the items (e.g., numeric odds ratio, percentage). Finally, univariate and multivariate logistic regression analyses of sociodemographic data were employed to identify those factors (independent variables) that could predict the classification of the participants into different subgroups (dependent variables), establish a thrombolytic decision-making model, and reveal the combined influence and mutual relationships of framing scenario, OTT interval, item formats, and sociodemographic factors (18).

### Statistical Analysis

Latent class analysis was performed first using Mplus 7.0 (21). Then, univariate and multiple logistic regression analyses (stepwise regression) were employed using SPSS19.0 (SPSS, Inc., Chicago, IL, USA). χ^2^ tests were performed to evaluate the differences in the rates of consent to thrombolysis among different OTT intervals and between different item formats. A significance level, from 0.05 to 0.00135, was set according to the partitioning of the χ^2^ method (α′ = α ÷ [*k* × (*k* − 1) ÷ 2 + 1], *K* = 9) (18).

## Results

### LCA of the Endorsement Rates of Thrombolysis

**Figure 1:**
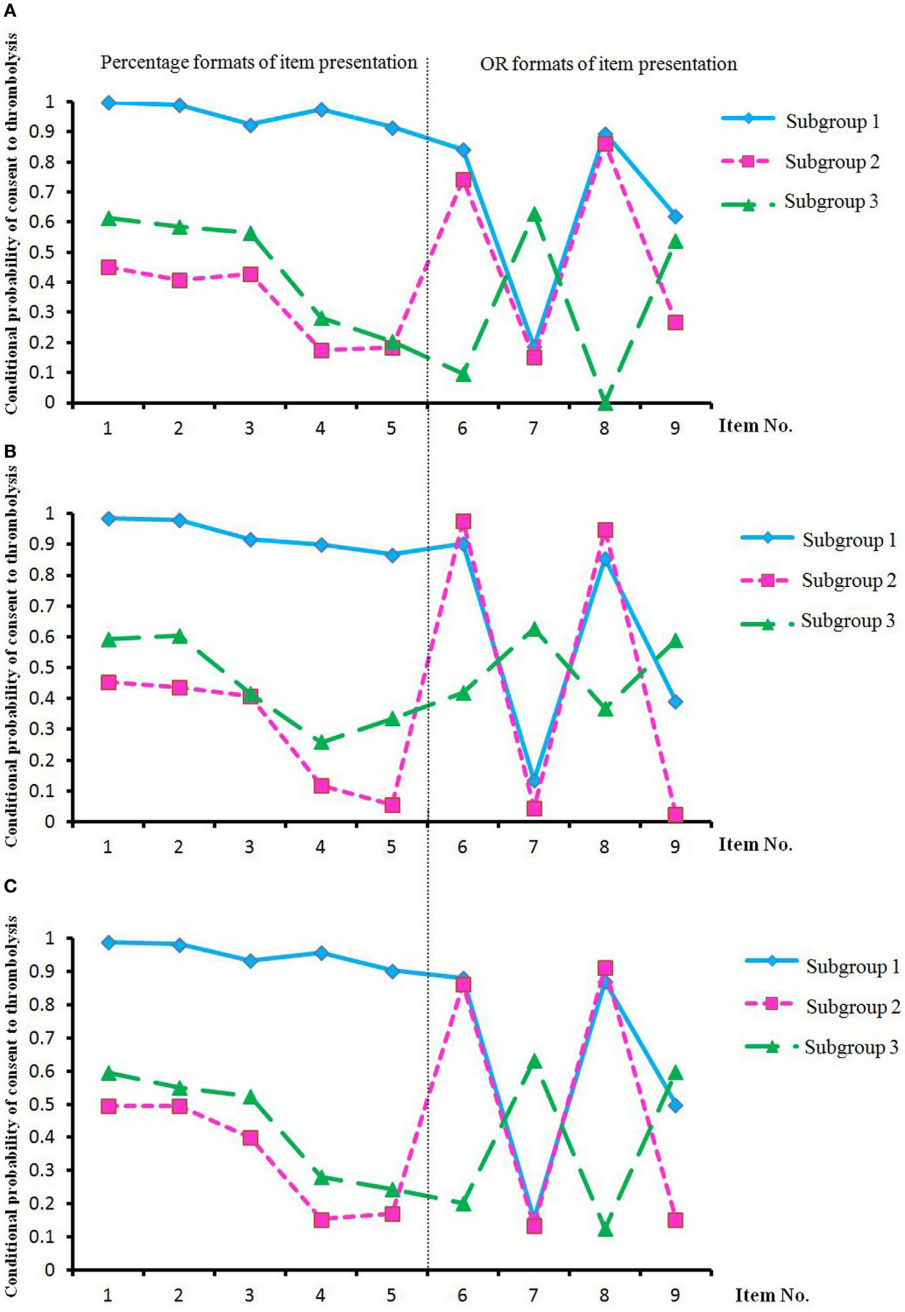
Conditional probability of consent to thrombolysis^a^ in three subgroups of participants^b^ in the negative-framing group **(A)**, the positive-framing group **(B)**, and both groups **(C)**. Abbreviations: subgroup 1, consent to thrombolysis subgroup; subgroup 2, objection to thrombolysis subgroup 1; subgroup 3, objection to thrombolysis subgroup 2; OR, odds ratio. ^a^The conditional probability of consent to thrombolysis for each item was calculated *via* latent class analysis (LCA), and high conditional probability indicated that the participants had more favorable attitudes toward thrombolysis, e.g., the participants in the consent to thrombolysis subgroup. ^b^The subgroup classification was determined based on LCA after randomization of positive or negative framing and the completion of the nine items.

Latent class analysis and the Lo–Mendell–Rubin likelihood ratio test of model fit (18, 22) indicated an optimal 3-subgroup model (Figure 1; Table 2). Under the percentage format, the participants in subgroup 1 preferred thrombolysis, whereas those in subgroups 2 and 3 preferred non-thrombolytic therapy. Under the numeric odds ratio (OR) format, the participants in subgroups 1 and 2 opted for thrombolysis for item 6 (format: numeric OR, OTT: 91–180 min, first presentation: thrombolytic therapy; i.e., item 6 refers to “There is no significant difference of survival rate between option A and option B. The results of follow-up at three months show that mild or no disability under option A is 1.52 times as much as that under option B. The rate of no parenchymal hemorrhage under option A is 0.12 times as much as that under option B.”) and item 8 (format: numeric OR, OTT: 181–270 min, first presentation: thrombolytic therapy) but not item 7 (format: numeric OR, OTT: 91–180 min, first presentation: non-thrombolytic therapy) or item 9 (format: numeric OR, OTT: 181–270 min, first presentation: non-thrombolytic therapy). These findings demonstrated an influence of the presentation order of the two options for items regardless of OTT interval. In contrast, the participants in subgroup 3 opted for thrombolysis for items 7 and 9 but not items 6 and 8, indicating that option order was influential but in the opposite direction to that observed for the other subgroups (Figure 1). Based on these findings, the three subgroups were designated the consent to thrombolysis subgroup, the objection to thrombolysis subgroup 1 (participants who were presented with numeric OR information and the thrombolytic therapy option first preferred thrombolytic therapy), and objection to thrombolysis subgroup 2 (participants who were presented with numeric OR information and the option of non-thrombolytic therapy first preferred thrombolytic therapy).

**Table 2:** Model fit statistics for different subgroup models in the negative- and positive-framing groups and both groups combined.

Framing type	No. of subgroups^a^	No. of parameters^b^	Log likelihood	AIC	BIC	Adjusted BIC	Entropy	LRT	Δdf	*p*
**Negative^c^**										
	1	9	−1,512.685	3,043.369	3,075.587	3,047.052	–	–	–	–
	2	19	−1,324.086	2,686.171	2,754.186	2,693.946	0.890	370.557	10	0.0000
	3	29	−1,274.892	2,607.784	2,711.596	2,619.651	0.848	96.655	10	0.0108
	4	39	−1,250.037	2,578.073	2,717.683	2,594.032	0.855	48.836	10	0.0842
	5	49	−1,225.354	2,548.708	2,724.115	2,568.759	0.895	40.807	10	0.2102

**Positive^d^**										
	1	9	−1,607.565	3,233.129	3,266.493	3,237.950	–	–	–	–
	2	19	−1,445.571	2,929.142	2,999.577	2,939.320	0.802	318.408	10	0.0000
	3	29	−1,383.979	2,825.958	2,933.464	2,841.493	0.794	121.063	10	0.0604
	4	39	−1,330.634	2,739.267	2,883.845	2,760.159	0.841	104.770	10	0.0016
	5	49	−1,317.347	2,732.694	2,914.343	2,758.943	0.812	26.115	10	0.1412

**Both^e^**										
	1	10	−3,537.114	7,094.227	7,137.613	7,105.868	–	–	–	–
	2	21	−3,192.339	6,426.678	6,517.789	6,451.124	0.921	–	–	–
	3	32	−3,078.092	6,220.184	6,359.019	6,257.434	0.879	–	–	–
	4	43	−3,015.038	6,116.076	6,302.636	6,166.131	0.929	–	–	–
	5	54	−2,983.679	6,075.357	6,309.642	6,138.217	0.918	–	–	–

*AIC, Akaike Information Criterion; BIC, Bayesian Information Criterion; LRT, Lo–Mendell–Rubin likelihood ratio test; LCA, latent class analysis*.

*^a^Many different classifications can be developed *via* LCA (e.g., 1-subgroup model, 2-subgroup model, and 3-subgroup model)*.

*^b^Concerning the fit measures (parsimony and goodness of fit), the model with fewest parameters (or subgroups), lowest BIC and AIC values and a significant *p* value for the LRT (<0.05), which should also be interpretable in medical practice, is preferable to the other models*.

*^c^After LCA in the negative-framing group, the 3-subgroup model was preferred according to the fit measures*.

*^d^After LCA in the positive-framing group, the 3-subgroup model was preferred according to the fit measures*.

*^e^After LCA in the negative- and positive-framing groups, the participants in both the negative and positive groups were combined, and their pooled responses to the items were again analyzed by LCA. The 3-subgroup model was found to be optimal according to the fit measures, which also corresponded to clinical practice*.

### χ^2^-Test of the Endorsement Rates of Thrombolysis across Different Framing Groups and Items

Significant differences in the constituent ratios of class probabilities were observed between the negative-framing and positive-framing groups, with more patients preferring thrombolysis in the positive-framing group (χ^2^ = 18.833, *p* < 0.001) (Table 3). In the negative-framing group, with the percentage format and regardless of framing, a greater rate of thrombolysis was observed for item 3 (format: percentage, OTT: 0–90 min, first presentation: non-thrombolytic therapy) than for item 4 (format: percentage, OTT: 0–90 min, first presentation: non-thrombolytic therapy) or 5 (format: percentage, OTT: 181–270 min, first presentation: non-thrombolytic therapy). In addition, in this group, a greater rate of thrombolysis was observed for item 9 than for item 7 (OR format). These findings indicated the influences of prognostic outcomes for different OTT intervals, formats of information, and framing messages on thrombolytic decision-making. The differences in the rates of thrombolysis between items 6 and 7 in both the negative-framing and positive-framing groups and between items 8 and 9 in the positive-framing group revealed the effects of option order for items and framing messages on consent to thrombolysis. The differences in the rates of thrombolysis between items 4 and 7 in both the negative- and positive-framing groups and between items 5 and 9 in the positive-framing group demonstrated the effects of information presentation format and message framing on consent to thrombolysis (Table 4).

**Table 3:** Conditional and classification probability of consent to thrombolysis in the negative-framing group, the positive-framing group, and both groups combined.

Conditional probability	Item no.	Negative-framing group			Positive-framing group			Both groups		
Presentation format of pros and cons		S1	S2	S3	S1	S2	S3	S1	S2	S3
**Percentage**										
	1	1.000	0.451	0.614	0.985	0.454	0.592	0.989	0.495	0.594
	2	0.991	0.408	0.585	0.978	0.436	0.604	0.982	0.495	0.550
	3	0.925	0.428	0.565	0.916	0.408	0.416	0.933	0.401	0.523
	4	0.976	0.175	0.283	0.899	0.119	0.258	0.958	0.153	0.281
	5	0.916	0.183	0.205	0.867	0.056	0.335	0.903	0.169	0.245

**Numeric OR**										
	6	0.842	0.742	0.097	0.903	0.976	0.419	0.880	0.863	0.203
	7	0.187	0.151	0.629	0.134	0.043	0.626	0.154	0.133	0.632
	8	0.896	0.861	0.000	0.854	0.949	0.368	0.869	0.912	0.126
	9	0.620	0.268	0.538	0.392	0.024	0.59	0.499	0.153	0.598

Classification probability (%)		38.1	32.4	29.5	45.1	27.2	27.7	40.6	33.6	25.8

*S1, consent to thrombolysis subgroup; S2, objection to thrombolysis subgroup 1; S3, objection to thrombolysis subgroup 2; OR, odds ratio*.

**Table 4:** χ^2^ test of rates of consent to thrombolysis between items in the negative group (upper right) and the positive group (bottom left).

OTT interval (min)			91–180	181–270	0–90	91–180	181–270	91–180	91–180	181–270	181–270
			
	Format of presentation		Percentage					OR			
		Item no.	1	2	3	4	5	6	7	8	9
91–180	Percentage	1		0.361 (*p* = 0.548)		19.123 (*p* < 0.001)					
181–270		2	0.006 (*p* = 0.940)				22.897 (*p* < 0.001)				
0–90		3				10.080 (*p* = 0.001)	17.506 (*p* < 0.001)				
91–180		4	30.846 (*p* < 0.001)		10.261 (*p* = 0.001)		1.039 (*p* = 0.308)	2.450 (*p* = 0.118)	23.870 (*p* < 0.001)		
181–270		5		31.546 (*p* < 0.001)	11.295 (*p* = 0.001)	0.027 (*p* = 0.870)				11.238 (*p* = 0.001)	0.045 (*p* = 0.832)
91–180	OR	6				50.626 (*p* < 0.001)			41.351 (*p* < 0.001)	0.640 (*p* = 0.424)	
91–180		7				44.409 (*p* < 0.001)		175.938 (*p* < 0.001)			16.515 (*p* < 0.001)
181–270		8					36.779 (*p* < 0.001)	1.607 (*p* = 0.205)			9.736 (*p* = 0.002)
181–270		9					14.362 (*p* < 0.001)		7.440 (*p* = 0.006)	92.959 (*p* < 0.001)	

*OTT, stroke onset to start of treatment; OR, odds ratio*.

*α′ = 0.00135*.

### Statistical Modeling by Logistic Regression Analysis

Univariate analyses were applied to identify inessential factors to exclude from the multivariate model (Appendix Table II in Supplementary Material). Multiple logistic regression analysis demonstrated that in contrast to the consent to thrombolysis subgroup, fewer stroke patients, self-ratings of poor health, a greater focus on health, less knowledge regarding infarction, a history of heart disease or drinking, an extroverted personality, and uncertain attitudes toward the importance of quality of life predicted the objection to thrombolysis subgroup 1, whereas negative-framing messages, knowledge workers, fewer stroke patients, a greater focus on health, less knowledge regarding infarction, an extroverted personality, and uncertain attitudes toward the importance of quality of life predicted the objection to thrombolysis subgroup 2 (Table 5).

**Table 5:** Multivariate analysis results of sociodemographic, health status, and attitude predictors for the classification of participants into subgroups.

Variable	Category	S2: S1^a^				S3: S1			
		*B*	SE	OR (95% CI)^b^	*p*	*B*	SE	OR (95% CI)	*p*
Frame type	Negative	−0.103	0.282	0.902 (0.519–1.568)	0.714	0.953	0.305	2.594 (1.426–4.717)	0.002
	Positive			1				1	

Occupation	Manual worker	−0.428	0.293	0.652 (0.367–1.157)	0.144	−0.752	0.324	0.472 (0.250–0.890)	0.020
	Knowledge worker			1				1	

Subject type	Stroke patient	−1.392	0.422	0.249 (0.109–0.568)	0.001	−0.953	0.461	0.386 (0.156–0.952)	0.039
	Stroke patient’s relative	−1.206	0.426	0.299 (0.130–0.690)	0.005	−0.820	0.467	0.441 (0.176–1.100)	0.079
	Non-stroke patient	−0.658	0.424	0.518 (0.226–1.189)	0.121	−0.262	0.449	0.770 (0.319–1.855)	0.560
	Non-stroke patient’s relative			1				1	

Health self-rating	Poor (very poor + poor)	1.467	0.483	4.338 (1.682–11.190)	0.002	0.991	0.529	2.693 (0.956–7.587)	0.061
	Intermediate	0.533	0.384	1.704 (0.803–3.616)	0.165	0.530	0.412	1.699 (0.758–3.807)	0.198
	Good (good + best)			1				1	

Focus on health	Less (not at all + less)	−1.012	0.414	0.363 (0.161–0.818)	0.014	−0.528	0.435	0.590 (0.252–1.383)	0.225
	Intermediate	−1.187	0.358	0.305 (0.151–0.616)	0.001	−1.160	0.402	0.313 (0.143–0.689)	0.004
	More (more + extremely)			1				1	

Knowledge regarding infarction	Less (not at all + no)	2.303	0.681	10.009 (2.632–38.057)	0.001	1.599	0.527	4.949 (1.760–13.911)	0.002
	Intermediate	2.105	0.677	8.208 (2.179–30.923)	0.002	0.494	0.534	1.639 (0.575–4.671)	0.355
	More (more + extremely)			1				1	

Anamnesis									
Heart disease	No	−1.374	0.422	0.253 (0.111–0.579)	0.001	−1.060	0.475	0.346 (0.137–0.879)	0.026
	Yes			1				1	

Drinking	No	−0.787	0.326	0.455 (0.240–0.863)	0.016	−0.436	0.354	0.646 (0.323–1.293)	0.218
	Yes			1				1	

Personality	Introverted	−1.385	0.288	0.250 (0.142–0.440)	<0.001	−1.179	0.311	0.307 (0.167–0.565)	<0.001
	Extroverted			1				1	

Attitude toward quality of life^c^	Strongly disagree + disagree	0.217	0.414	1.242 (0.552–2.795)	0.601	−0.620	0.550	0.538 (0.183–1.581)	0.260
	Uncertain	1.292	0.515	3.638 (1.327–9.974)	0.012	1.667	0.510	5.296 (1.950–14.381)	0.001
	Strongly agree + agree			1				1	

Constant		1.626	0.931		0.081	1.071	0.890		0.229
Pseudo-*R*^2d^	0.366								
Model *p*^e^	<0.001								

*S1, consent to thrombolysis subgroup; S2, objection to thrombolysis subgroup 1; S3, objection to thrombolysis subgroup 2; OR, odds ratio; *B*, unstandardized *B* coefficient; SE, standard error (*B*); CI, confidence interval*.

*^a^The consent to thrombolysis subgroup acted as the reference subgroup*.

*^b^Multiple logistic regression models were generated using backward stepwise selection*.

^c^Participant attitude toward the statement “One’s quality of life is more important than his or her lifespan.”

*^d^Pseudo-*R*^2^ was calculated an index of model goodness of fit, and the pseudo-*R*^2^ value (0.366) indicated that the factors in the model accounted for 36.6% of the variation in the prediction of assignment of subgroups, suggesting adequate goodness of fit of the model*.

*^e^Model *p* value indicates the significance of model (*p* value < 0.05)*.

The multivariate model was established as follows:

Y1 = 0
Y2 = 1.626–1.392*Stroke patients − 1.206*Stroke patients’ relatives + 1.467*Poor health self-rating − 1.012*Less focus on health − 1.187*Intermediate focus on health + 2.303*Less knowledge about infarction + 2.105*Intermediate knowledge about infarction − 1.374*No heart disease − 0.787*No drinking − 1.385*Introverted + 1.292* uncertain opinion about whether “One’s quality of life is more important.”
Y3 = 1.071 + 0.953*Frame type − 0.752*Occupation − 0.953*Stroke patients − 1.160* Intermediate focus on health + 1.599*Less knowledge about infarction − 1.060*No heart disease − 1.179*Introverted + 1.667*uncertain opinion about whether “One’s quality of life is more important.”

The intended probabilities of classification into different subgroups for each person could be calculated according to the following equations (e = 2.71828):

Probability of classification into C1 = e^Y1^/[e^Y1^ + e^Y2^ + e^Y3^]
Probability of classification into C2 = e^Y2^/[e^Y1^ + e^Y2^ + e^Y3^]
Probability of classification into C3 = e^Y3^/[e^Y1^ + e^Y2^ + e^Y3^].

## Discussion

Our data confirm our initial hypothesis that thrombolytic decision-making varies by prognostic information for different OTT intervals. As mentioned earlier, for the AIS patients, earlier treatment is closely related to greater proportional benefits, higher cost-effectiveness and improvement in quality of life. Specifically, when using the percentage format, the results indicated that participants who were presented with prognostic outcomes during the OTT interval of 0–90 min were more likely to accept thrombolytic therapy than were patients presented with outcomes during OTT intervals of 91–180 or 181–270 min. These findings indicate that it is necessary to present patients with prognostic outcomes specific to their OTT interval to obtain consent to thrombolysis. Our finding that thrombolysis was more favored in the positive-framing scenario than in the negative-framing scenario are consistent with other classic findings showing that the attractiveness of risk-seeking options (such as surgery) is substantially greater than that of risk-aversion options (like radiation therapy) when the problem is framed positively (in terms of the probability of living) rather than negatively (i.e., the probability of dying). A possible explanation for this phenomenon is that treatment and/or attribute framing are more preferred in the positive scenario than in the negative scenario (18).

This study corroborates our second hypothesis that choices regarding thrombolysis are modified by not only prognostic information for different OTT intervals but also message framing, presentation format, and participant characteristics, values, and preferences. The differences in thrombolysis rates between the percentage mode and OR mode of item presentation in the OTT interval of 181–270 min in different framing messages corroborate the view that thrombolytic decision-making is influenced not only by the framing effect but also by the mode of presentation of therapeutic outcomes. According to the logistic regression analyses, in contrast to the consent to thrombolysis subgroup, fewer stroke patients, a greater focus on health, less knowledge regarding infarction, an extroverted personality, and uncertain attitudes toward the importance of quality of life predicted the objection to thrombolysis subgroups 1 and 2, which indicates that participants with these factor levels are insensitive to the information presented in percentage mode. Furthermore, our data also indicate that the order in which therapies are presented for items greatly affects perceptual judgments of medical decision-making, especially under the OR mode. According to the modeling results, in contrast to the consent to thrombolysis subgroup, self-ratings of poor health and a history of heart disease or drinking predicted the objection to thrombolysis subgroup 1, in which participants are sensitive to thrombolytic therapy as the first presentation of therapy in the items in OR mode, whereas negative-framing messages and knowledge workers predicted the objection to thrombolysis subgroup 2, in which participants are sensitive to non-thrombolytic therapy as the first presentation of therapy in the items in OR mode. These findings suggest that physicians should decide the order of presentation of therapies according to the classification of patients/proxies to promote consent to thrombolysis (Figure 1).

The identification of distinctive subgroups of participants by LCA and of predictors of internal factors for these three subgroups by logistic regression facilitate the establishment of multivariable models of thrombolytic decision-making. Based on the multivariate decision model, physicians can help patients *via* an individualized decision-making (IDM) process (18) in which different patients are presented with different thrombolysis-related items in accordance with their individual characteristics to shorten treatment delay and increase their likelihood of choosing thrombolysis. For example, the patient’s individual variable values can be entered into the equation, the positive- or negative-framing messages can be set, and then the probability of the patient’s assignment to each subgroup can be calculated. Because we have enhanced our understanding of the conditional probability of consent to thrombolysis with the different items within each subgroup, we can note in which item the patient would most prefer thrombolytic or non-thrombolytic therapy. This process, which we call IDM, can be used to guide clinicians toward maximizing an individual’s consent to thrombolysis. In contrast, shared decision-making (SDM) is a patient-centered process of collaboration between clinicians and patients and is currently viewed as fundamental to safe and effective healthcare (23). SDM is criticized for its abuse in unsuitable patients who make irrational decisions (24) and is considered inferior to authoritarian decision-making (ADM), which emphasizes the responsibilities of clinicians (25). To the best of our knowledge, IDM can eliminate conflicts between SDM and ADM and account for both clinicians’ responsibilities and patients’ values and preferences during medical decision-making (18). In contrast, to convey unbiased information to stroke patients/proxies in a traditional manner, physicians should use diverse presentation formats, both of framing information and prognostic messages for different OTT intervals, to explain the risks and benefits of thrombolysis. This process might require considerable additional time beyond the treatment delay appropriate for thrombolysis.

Recently, a presumption of consent to intravenous thrombolytic therapy for stroke has recently been supported by professional societies (8). This presumption has been further supported by empirical studies that favored the application of thrombolysis for stroke in emergency circumstances under the presumption of consent (26, 27). However, it is suggested that thrombolysis for stroke is “autonomy saving” and not “life saving” and that it carries a risk of symptomatic intracranial hemorrhage, which implies that informed consent to thrombolytic therapy should be obtained (28). Furthermore, it has been suggested that thrombolytic decision-making does not involve science *per se* but rather the domains of law, ethics, and policies (13). However, our findings present an argument for applying science to a process that while steeped in ethics may benefit from more scientifically rigorous analysis and application.

Although the process of obtaining informed consent can have clinical consequences, few studies are available that indicate the extent to which the consent process can affect outcome or that can instruct physicians on how best to promote thrombolysis in time (29). In this study, both LCA and logistic regression were employed to explore patient preferences for thrombolysis and to establish a decision model for thrombolytic therapy. LCA was used to identify different groups of participants according to their response patterns for thrombolysis (30). LCA is similar to cluster analytic methods but is more appropriate for binary data (31). LCA permitted analysis of the comprehensive effects of framing message, mode of item presentation, therapeutic outcomes with different OTTs, and individual characteristics upon thrombolytic decision-making. The presented method for establishing a thrombolytic decision model through the combination of LCA and logistic regression can inform the establishment of decision models in other clinical domains, such as in the promotion of disease prevention and screening.

### Limitations

(1) The number of participants was small. (2) There was an excessive number of items for the participants, especially for the elderly participants. (3) We did not classify stroke patients into different subtypes, otherwise the overall discussion would benefit from knowing; it would have been informative to know how many respondents were actual stroke patients. (4) Participant economic status and health insurance, which are potential factors influencing clinical decision-making, were not recorded. For example, the cost of thrombolytic drugs (at least $870) is not covered by insurance in Beijing unless treatment is initiated within 180 min, which detracts from thrombolytic treatment. (5) This study did not address the intra-arterial thrombolytic/embolectomy approach, which should be studied in future research. (6) Other factors that influence thrombolysis but that were not addressed here warrant investigation, including personality traits, patient disability, decision-making style (individual vs. group), the order of information, thinking pattern (intuitive vs. analytical), and cultural traits.

## Conclusion

Choices regarding thrombolysis are modified by not only prognostic information for different OTT intervals but also message framing, presentation format, and sociodemographic characteristics. To facilitate consent to thrombolysis, physicians should convey prognostic information to patients/proxies on the basis of patient OTT interval and should order the presentation of therapies according to the classification of patients/proxies. IDM might be an optimal strategy to increase the selection of thrombolysis; this strategy challenges traditional approaches with respect to thrombolytic decision-making. The presented method for establishing a thrombolytic decision model through the combination of LCA and logistic regression can inform the establishment of decision models in other clinical domains, such as in the promotion of disease prevention and screening.

## Ethics Statement

This study was carried out in accordance with the recommendations of the PLA Army General Hospital ethics committee with written informed consent from all subjects. All subjects gave written informed consent in accordance with the Declaration of Helsinki. The protocol was approved by the PLA Army General Hospital ethics committee.

## Author Contributions

JG and YH contributed to the study design and participated in performing the experiments. HL and JG contributed to the study design and analyzed the data. YZ searched the literature, participated in data collection, and wrote the manuscript. HG, WW, and JL participated in performing the experiments and in data collection and interpretation. YZ prepared the psychological assessment/materials/analysis tools and revised the manuscript. YH supervised the study.

## Conflict of Interest Statement

The authors declare that the research was conducted in the absence of any commercial or financial relationships that could be construed as a potential conflict of interest. The reviewer MS and handling Editor declared their shared affiliation.
