# Supplementary material for: Choices Regarding Thrombolysis Are Modified by the Way to Transfer the Messages
Source: Front Neurol. 2017 Nov 7;8:589. doi: 10.3389/fneur.2017.00589 (PMC5683066; doi:10.3389/fneur.2017.00589)
Supplement: Supplementary file 1 [file data_sheet_1.zip › Appendix-2016-10.docx]

**Appendixes**

**Appendix Materials**

**AppendixTables**

Appendix Table Ⅰ

Socio-demographic, health statue / knowledge, psychological characteristics, and attitudes data of the participants in the negative framing group and the positive framing group

Appendix Table II

Univariate analysis of sociodemographic, health status, and attitude predictors for the classification of participants into subgroups

**Appendix References**

**Appendix Materials**

***Instructions*** Participants received a letter indicating that they would respond to a series of questions, which they were urged to answer carefully and truthfully as all choices were acceptable and rational.

***Numeracy measure*** Participants were required to complete the Numeracy Scale which consisted of 3 general numeracy scale items and 8 expanded numeracy scale items:

Here is a short quiz. Please try to complete the following questions:

How many times would a coin land heads-up when tossed 1000 times?

Answer: (this is a practice question, and the answer will be provided to the participants)

1. Imagine that we roll a fair, six-sided die 1,000 times. Out of the 1,000 rolls, how many times do you think the die would come up even (2, 4, or 6)? ___

2. In a public welfare lottery, the chance of winning a ¥10·00 prize is 1%. What is your best guess of the number of people winning a ¥10.00 prize if 1,000 people each buy a single ticket for the public welfare lottery? ___

3. In a lottery draw at a supermarket, the chance of winning an [electromobile](http://www.iciba.com/electrombile) is 1 in 1,000. What percentage of the tickets in the lottery draw will win an [electromobile](http://www.iciba.com/electrombile)? ___

4. Which of the following numbers represents the greatest risk of getting a disease?

___ 1 in 100; ___ 1 in 1000; ___ 1 in 10 (put a tick” √” next to the answer )

5. Which of the following numbers represents the greatest risk of getting a disease?

___ 1%; ___ 10%; ___ 5% (put a tick” √” next to the answer)

6. If Person A’s risk of having an infarction is 1% in ten years, and person B’s risk is double that of A’s, what is B’s risk in ten years? ___

7. If Person A’s risk of having an infarction is 1 in 100 in ten years, and person B’s risk is double that of A’s, what is B’s risk in ten years? ___

8. If the chance of having an infarction is 10% in a population, how many people would be expected to get the disease:

A. Out of 100? ___

B. Out of 1000? ___

9. If the chance of having an infarction is 20 out of 100, this would be the same as having a ___% chance of getting the disease.

10. The chance of getting a viral infection is .0005. Out of 10,000 people, about how many of them are expected to become infected? ___

(This scale is modified from Lipkus’s study[^1^](#_ENREF_1))

***Different formats of thrombolytic information presentation*** The medical scenario was set in "acute cerebral infarction", and thrombolytic therapy was presumed to be suitable for all the patients or their next of kin who were asked to choose between two options (thrombolytic therapy or not) in 9 different simulated hospitals (see Table II). In the positive framing scenario, both the information about each option was described in terms of rates of survival, mild disability (or no disability), and no parenchymal haemorrhage, while in the negative framing scenario, they were described by rates of mortality, severe disability, and parenchymal haemorrhage. The formats of thrombolytic information presentation for prognosis included percentage and Odds Ratios (OR). All the data are adapted from the findings of large randomized controlled trials and observational studies with different onset to start of treatment.

***Socio-demographic, health & attitude questionnaire*** The questionnaire contained not only socio-demographic questions (age, gender, nationality, marital status, etc.) but also participants’ health and attitude items:

**Cerebral infarction is an ischaemic cerebrovascular disease that poses a great threat to human health. Suppose you or your relatives are facing the threat of acute cerebral infarction:**

**In Hospital No. 1001 (Note: 1001 is the code for a hospital, as are 1002, 1004, etc.), two options of treatment are offered to the patients and their families:**

Option 1: The survival rate is 84.4%. Follow-ups in three months show that 28.9% of patients have mild disability or no disability.

Option 2: The survival rate is 83.2%. Follow-ups in three months show that 41.9% of patients have mild disability or no disability.

Which option will you prefer?

**In Hospital No. 1002, two options of treatment are offered to the patients and their families:**

Option 3: The survival rate is 89.9%. Follow-ups in three months show that 37.7% of the patients have mild or no disability.

Option 4: The survival rate is 89.0%. Follow-ups in three months show that 44.6% of the patients have mild or no disability.

Which option will you prefer?

**In Hospital No. 1004, two options of treatment are offered for the patients and their families:**

Option 7: The survival rate is 79.5%. Follow-ups in three months show that 29.1% of patients have mild disability or no disability. Additionally, 100% of patients don’t suffer from parenchymal haemorrhage.

Option 8: The survival rate is 81.4% Follow-ups in three months show that 41.6% of patients have mild disability or no disability. Additionally, 96.9% of patients don’t suffer from parenchymal haemorrhage.

Which option will you prefer?

**In Hospital No. 1005, two options of treatment are offered for the patients and their families:**

Option 9: The survival rate is 84.4%. Follow-ups in three months show that 28.9% of patients have mild disability or no disability. Additionally, 99.0% of patients don’t suffer from parenchymal haemorrhage.

Option 10: The survival rate is 83.2%. Follow-ups in three months show that 41.9% of patients have mild disability or no disability. Additionally, 94.4% of patients don’t suffer from parenchymal haemorrhage.

Which option will you prefer?

**In Hospital No. 1006, two options of treatment are offered to the patients and their families:**

Option 11: The survival rate is 89.9%. Follow-ups in three months show that 37.7% of the patients have mild or no disability. Additionally, 98.8% of the patients don’t suffer from parenchymal haemorrhage.

Option 12: The survival rate is 89.0%. Follow-ups in three months show that 44.6% of the patients have mild or no disability. Additionally, 95.7% of the patients don’t suffer from parenchymal haemorrhage.

Which option will you prefer?

**Anonymous Demographic and Health Status Questionnaire**

**Tips:** Please fill in the answer directly on the line or place a tick “√” in the brackets next to the answer you would like to choose.

**1. Age:** ____years; **2. Gender:** _____; **3. nationality:** _____

**4. Present and past occupation:** __________________ (please fill in on the line)

**5. Education background:** ________(such as primary school, high school, college, Master’s or Doctoral degree).

**6. You might be characterized as:** Introverted ( ) or Extroverted ( )

**7. Religion or Belief:** None ( ); Yes ( );

if yes, please specify:________________________

**8. Marital status:**

① Single ( ) ② Boyfriend or Girlfriend ( ) ③ Married ( ) ④ Divorced ( ) ⑤ Widowed ( )

**9. How often you usually see a physician (including hospitalization):**

① less than once a year ( ) ② once a year ( ) ③ once / 6 months ( ) ④ 1-2 times / 3 months( ) ⑤ 1-2 times / month ( )

**10. Overall assessment of your current health:**

① very poor ( ) ② poor ( ) ③ intermediate ( ) ④ good ( ) ⑤ best ( )

**11. How well do you pay attention to your health?**

① Not at all ( ) ② less ( ) ③ intermediate ( ) ④ more ( ) ⑤ a great deal ( )

**12. Do you smoke cigarettes?** Yes ( ); No ( );

if yes, how many years have you smoked? _______

If yes, how many cigarettes do you smoke a day?_______

**13. Do you drink alcohol?** Yes ( ); No ( );

if yes, for how many years have you been drinking? _______

if yes, how much alcohol do you consume a day? _______

**14. Do you have immediate family members (such as parents and siblings related by blood) suffering from acute cerebral infarction?**

① No ( ); ② Yes ( );

**15. Do you currently have or have you ever had high blood pressure?**

① no ( ); ② Yes ( );

**16. Do you currently have or have you ever had hyperlipidaemia (high blood lipid levels)?**

① No ( ); ② Yes ( );

**17. Do you currently receive or have you ever received lipid-lowering medications?**

① No ( ); ② Yes ( );

**18. Do you currently have or have you ever had heart disease?**

① No ( ); ② Yes ( );

**19. How much do you know about "acute cerebral infarction" and its treatment:**

① Nothing at all ( ) ② no ( ) ③ a moderate amount ( ) ④ more ( ) ⑤ a great deal ( )

**20. What is your identity?**

① inpatient ( ) ② immediate family members of inpatients ( )

**21. Have you ever been diagnosed with "cerebral infarction"?**

① Yes ( ); ② No ( ); if yes, which type of infarction you suffer from?

① acute cerebral infarction ( ) ② lacunar infarction ( ) ③old infarction ( )④ infarction sequelae ( ) ⑤ Other__________________ (specify on the line)

**22. Do you agree with the following viewpoints?**

**1) If necessary, thrombolytic therapy should be recommended to friends and families**

① strongly disagree ( ) ② disagree ( ) ③ uncertain ( )

④ agree ( ) ⑤ strongly agree ( )

**2) The doctor should recommend thrombolytic therapy**

① strongly disagree ( ) ② disagree ( ) ③ uncertain ( )

④ agree ( ) ⑤ strongly agree ( )

**3) Authorities should enact laws to mandate that thrombolytic treatment should be given to the patients who are considered to be suitable for such treatment.**

① strongly disagree ( ) ② disagree ( ) ③ uncertain ( )

④ agree ( ) ⑤ strongly agree ( )

**4) Patients and their families should be allowed to refuse thrombolytic therapy**

① strongly disagree ( ) ② disagree ( ) ③ uncertain ( )

④ agree ( ) ⑤ strongly agree ( )

**5) One’s quality of life is more important than his or her lifespan.**

① strongly disagree ( ) ② disagree ( ) ③ uncertain ( )

④ agree ( ) ⑤ strongly agree ( )

**Cerebral infarction is an ischaemic cerebrovascular disease that poses a great threat to human health. Suppose you or your relatives are facing the threat of acute cerebral infarction:**

**In Hospital No. 4001, two options of treatment (Option A and B) are offered for the patients and their families:**

There is no significant difference of survival rate between Option A and Option B. The results of follow-up at three months show that mild or no disability under Option A is 1.52 times as much as that under Option B. The rate of no parenchymal haemorrhage under Option A is 0.12 time as much as that under Option B.

Which option will you prefer?

**In Hospital No. 4002, two options of treatment (Option C and D) are offered for the patients and their families:**

There is no significant difference of survival rate between Option C and Option D. The results of follow-up at three months show that mild or no disability under Option C is 0.66 time as much as that under Option D. The rate of no parenchymal haemorrhage under Option C is 8.23 times as much as that under Option D.

Which option will you prefer?

**In Hospital No. 4003, two treatment options (Options E and F) are offered to patients and their families:**

There is no significant difference in survival rates between Option E and Option F. The results of follow-up at three months show that the possibility of mild or no disability under Option E is 1.32 times greater than under Option F. The rate of no parenchymal haemorrhage under Option E is 0.28 times that under Option F.

Which option will you prefer?

**In Hospital No. 4004, two treatment options (Option G and H) are offered to the patients and their families:**

There is no significant difference in survival rates between Option G and Option H. The results of follow-up at three months show that the rate of mild or no disability under Option G is 0.76 times that under Option H. The possibility of no parenchymal haemorrhage under Option G is 3.61 times greater than that under Option H.

Which option will you prefer?

***Psychological characteristics*** The SCL-90-R was used to measure the psychological state of participants. It is a validated and reliable self-rated scale which is usually chosen to assess 10 postulated factors: depression, somatisation, anxiety, phobic anxiety, obsessive-compulsive, interpersonal sensitivity, hostility, psychoticism, paranoid ideation, and additional factors (sleep and concentration difficulties). The inventory comprised 90 items with a rating scale with 5 degrees of severity (from “not at all” to “extremely”) according to his or her mood state over the past week. We used the Chinese translation by Wang[^2^](#_ENREF_2).

**Appendix Tables**

**Appendix Table Ⅰ Socio-demographic, health statue / knowledge, psychological characteristics, and attitudes data of the participants in the negative framing group and the positive framing group**

|  | **Negative**  **(n=265)** | **Positive**  **(n=301)** | ***p*** |
| --- | --- | --- | --- |
| **Socio-demographic Characteristics** |  |  |  |
| **Gender, *n* (%)** |  |  | 0.083 |
| Male | 152 | 154 |  |
| Female | 104 | 142 |  |
| **Education** |  |  | 0.397 |
| Primary school | 44 | 34 |  |
| Junior middle school | 27 | 38 |  |
| High school | 109 | 126 |  |
| College | 65 | 79 |  |
| Postgraduate | 3 | 5 |  |
| **Age** |  |  | 0.237 |
| 18–39 y | 63 | 57 |  |
| 40–59 y | 109 | 126 |  |
| >60 y | 81 | 109 |  |
| **Nationality** |  |  | 0.253 |
| Han | 251 | 277 |  |
| others | 14 | 23 |  |
| **Marital status** |  |  | 0.856 |
| married/living with partner | 229 | 261 |  |
| unmarried/divorced/widowed | 34 | 37 |  |
| **Religious belief** |  |  | 0.339 |
| with religious belief | 29 | 26 |  |
| Without religious belief | 233 | 274 |  |
| **Occupation** |  |  | 0.128 |
| [manual worker](http://www.iciba.com/manual_workers) | 156 | 155 |  |
| Knowledge worker | 82 | 108 |  |
| **Subject types** |  |  | 0.102 |
| Stroke patients | 75 | 106 |  |
| Stroke patients’ relatives | 65 | 81 |  |
| Non-stroke patients | 68 | 69 |  |
| Non-stroke patients’ relatives | 57 | 45 |  |
| **Numeracy** |  |  | 0.536 |
| Low score(<10) | 116 | 124 |  |
| High score (10-11) | 149 | 177 |  |
| **health statue and knowledge /attention** |  |  |  |
| **Check-up frequency (including hospitalization)** |  |  | 0.700 |
| <once 6 months | 217 | 241 |  |
| once 6 months | 25 | 35 |  |
| **>** once 6 months | 21 | 23 |  |
| **Health self-rating** |  |  | 0.073 |
| Poor (very poor+poor) | 46 | 63 |  |
| Intermediate | 157 | 189 |  |
| Good (good+best) | 62 | 48 |  |
| **Focus on health** |  |  | 0.299 |
| Less (not at all + less) | 76 | 78 |  |
| Intermediate | 122 | 130 |  |
| More (more + extremely) | 63 | 89 |  |
| **Anamnesis** |  |  |  |
| Cerebral infarction |  |  | 0.738 |
| Yes | 102 | 120 |  |
| No | 163 | 181 |  |
| hypertension |  |  | 0.198 |
| Yes | 90 | 118 |  |
| No | 174 | 182 |  |
| hyperlipidemia |  |  | 0.053 |
| Yes | 76 | 109 |  |
| No | 189 | 191 |  |
| heart disease |  |  | 0.155 |
| Yes | 32 | 49 |  |
| No | 232 | 251 |  |
| smoking |  |  | 0.063 |
| Yes | 96 | 87 |  |
| No | 169 | 214 |  |
| drinking |  |  | 0.984 |
| Yes | 66 | 75 |  |
| No | 197 | 223 |  |
| stroke in relatives |  |  | 0.623 |
| Yes | 65 | 84 |  |
| No | 146 | 171 |  |
| **Knowledge about infarction** |  |  | 0.785 |
| Less (not at all +no) | 113 | 126 |  |
| Intermediate | 124 | 147 |  |
| More (more+extremely) | 24 | 23 |  |
| **Psychological characteristics and attitudes** |  |  |  |
| **Personality** |  |  | 0.358 |
| Introverted | 124 | 158 |  |
| Extroverted | 118 | 128 |  |
| **Attitude toward quality of life “ One’s quality of life is more important”** |  |  | 0.103 |
| Disagree (Strongly disagree + disagree) | 20 | 37 |  |
| Uncertain | 25 | 34 |  |
| Agree (Strongly agree + agree) | 218 | 226 |  |
| **SCL-90-R** |  |  |  |
| Total average | 1.44 ±0.48 | 1.50 ±0.47 | 0.213 |
| Number of positive items | 24.90 ±22.29 | 28.10 ±21.85 | 0.102 |
| Number of negative items | 65.10 ±22.29 | 61.90 ±21.85 | 0.102 |
| Average of positive items | 2.44 ±0.38 | 2.46 ±0.42 | 0.595 |
| Somatisation | 1.57 ±0.53 | 1.62 ±0.54 | 0.310 |
| Obsessive-compulsive | 1.61 ±0.63 | 1.67 ±0.63 | 0.271 |
| Interpersonal sensitivity | 1.43 ±0.53 | 1.49 ±0.54 | 0.159 |
| Depression | 1.45 ±0.59 | 1.50 ±0.54 | 0.293 |
| Anxiety | 1.36 ±0.51 | 1.42 ±0.49 | 0.166 |
| Hostility | 1.47 ±0.54 | 1.57 ±0.62 | **0.047** |
| Phobic anxiety | 1.33 ±0.53 | 1.35 ±0.51 | 0.722 |
| Paranoid ideation | 1.36 ±0.50 | 1.41 ±0.55 | 0.290 |
| Psychoticism | 1.35 ±0.49 | 1.40 ±0.49 | 0.294 |
| Additional factors | 1.58 ±0.61 | 1.59 ±0.56 | 0.771 |

**Appendix Table Ⅱ Univariate analysis of sociodemographic, health status, and attitude predictors for the classification of participants into subgroups**

| **Variables** | **Category** | **S1** | **S2** | **S3** |  | **OR (95% CI)** | ***p*** |
| --- | --- | --- | --- | --- | --- | --- | --- |
|  |  | **N (%)** | **N (%)** | **N (%)** | **S1 as a reference** | |  |
| **Frame type** | Negative | 102(44.3) | 73(38.4) | 90(61.6) | *2:1^a^* | 0.783(0.529-1.158) | 0.220 |
|  | Positive | 128(55.7) | 117(61.6) | 56(38.4) | *3:1^b^* | 2.017(1.321-3.079) | **0.001** |
| **Gender** | Male | 122(56.0) | 107(56.6) | 77(53.1) | *2:1* | 1.027(0.693-1.521) | 0.895 |
|  | Female | 96(44.0) | 82(43.4%) | 68(46.9) | *3:1* | 0.891(0.584-1.359) | 0.592 |
| **Education** | Primary/Junior middle school | 64(29.5) | 47(26.6) | 32(23.5) | *2:1* | 0.764(0.445-1.312) | 0.329 |
|  | High school | 101(46.5) | 80(45.2) | 54(39.7) | *2:1* | 0.824(0.506-1.340) | 0.435 |
|  | College/ Postgraduate | 52(24.0) | 50(28.2) | 50(36.8) | *3:1* | 0.520(0.293-0.924) | **0.026** |
|  |  |  |  |  | *3:1* | 0.556(0.334-0.926) | **0.024** |
| **Age** | 18–39 y | 44(20.5) | 47(25.3) | 29(20.1) | *2:1* | 1.353(0.794-2.305) | 0.266 |
|  | 40–59 y | 95(44.2) | 79(42.5) | 61(42.4) | *2:1* | 1.053(0.671-1.654) | 0.821 |
|  | >60 y | 76(35.3) | 60(32.3) | 54(37.5) | *3:1* | 0.928(0.517-1.664) | 0.801 |
|  |  |  |  |  | *3:1* | 0.904(0.562-1.452) | 0.676 |
| **Nationality** | Han | 219(95.6) | 173(91.1) | 136(93.2) | *2:1* | 0.465(0.208-1.041) | 0.062 |
|  | others | 10(4.4) | 17(8.9) | 10(6.8) | *3:1* | 0.621(0.252-1.531) | 0.301 |
| **Marital status** | unmarried/divorced/widowed | 22(9.6) | 27(14.4) | 22(15.2) | *2:1* | 1.570(0.862-2.860) | 0.140 |
|  | married/living with partner | 206(90.4) | 161(85.6) | 123(84.8) | *3:1* | 1.675(0.890-3.150) | 0.110 |
| **Religious belief** | with religious belief | 14(6.1) | 24(12.7) | 17(11.7) | *2:1* | 2.223(1.116-4.431) | **0.023** |
|  | Without religious belief | 214(93.9) | 165(87.3) | 128(88.3) | *3:1* | 2.030(0.968-4.257) | 0.061 |
| **Occupation** | [manual worker](http://www.iciba.com/manual_workers) | 148（71.2） | 90（53.3） | 73（58.9） | *2:1* | 0.462(0.302-0.707) | **<0.001** |
|  | knowledge worker | 60（28.8） | 79（46.7） | 51（41.1） | *3:1* | 0.580(0.364-0.926) | **0.022** |
| **Subject types** | Stroke patients | 78(33.9) | 63(33.2) | 40(27.4) | *2:1* | 0.720(0.405-1.280) | 0.263 |
|  | Stroke patients’ relatives | 73(31.7) | 45(23.7) | 28(19.2) | *2:1* | 0.550(0.302-1.000) | **0.050** |
|  | Non-stroke patients | 46(20.0) | 45(23.7) | 46(31.5) | *2:1* | 0. 873(0.468-1.628) | 0.668 |
|  | Non-stroke patients’ relatives | 33(14.3) | 37(19.5) | 32(21.9) | *3:1* | 0.529(0.285-0.981) | **0.043** |
|  |  |  |  |  | *3:1* | 0.396(0.206-0.760) | **0.005** |
|  |  |  |  |  | *3:1* | 1.031(0.546-1.946) | 0.924 |
| **Numeracy** | Low score（≤ 9） | 87(37.8) | 86(45.3) | 67(45.9) | *2:1* | 1.394(0.915-2.123) | 0.122 |
|  | High score (10-11) | 143(62.2) | 104(54.7) | 79(54.1) | *3:1* | 1.657(0.620-4.431) | 0.314 |
| **Check-up frequency** | <once 6 months | 209(90.9) | 137(72.5) | 112(78.3) | *2:1* | 0.236(0.107-0.521) | **<0.001** |
|  | once 6 months | 12(5.2) | 27(14.3) | 21(14.7) | *2:1* | 0.810(0.292-2.429) | 0.686 |
|  | > once 6 months | 9(3.9) | 25(13.2) | 10(7.0) | *3:1* | 0.482(0.190-1.222) | 0.124 |
|  |  |  |  |  | *3:1* | 1.575(0.501-4.956) | 0.437 |
| **Health self-rating** | Poor (very poor+poor) | 28(12.2) | 53(28.0) | 28(19.2) | *2:1* | 1.793(0.940-3.422) | 0.076 |
|  | Intermediate | 166(72.2) | 98(51.9) | 82(56.2) | *2:1* | 0.559(0.333-0.941) | **0.028** |
|  | Good (good+best) | 36(15.7) | 38(20.1) | 36(24.7) | *3:1* | 1.000(0.497-2.011) | 1.000 |
|  |  |  |  |  | *3:1* | 0.494(0.290-0.841) | 0.009 |
| **Focus on health** | Less (not at all + less) | 55(24.4) | 51(27.1) | 48(33.1) | *2:1* | 0.520(0.299-0.905) | **0.021** |
|  | Intermediate | 133(59.1) | 71(37.8) | 48(33.1) | *2:1* | 0.299(0.182-0.491) | **<0.001** |
|  | More (more + extremely) | 37(16.4) | 66(35.1) | 49(33.8) | *3:1* | 0.659(0.370-1.173) | 0.493 |
|  |  |  |  |  | *3:1* | 0.273(0.159-0.467) | **<0.001** |
| **Anamnesis** |  |  |  |  |  |  |  |
| Cerebral infarction | No | 132(57.4) | 117(61.6) | 95(65.1) | *2:1* | 1.190(0.804-1.761) | 0.385 |
|  | Yes | 98(42.6) | 73(38.4) | 51(34.9) | *3:1* | 1.383(0.901-2.124) | 0.139 |
| Hypertension | No | 163(70.9) | 108(57.1) | 85(58.6) | *2:1* | 0.548(0.366-0.822) | **0.004** |
|  | Yes | 67(29.1) | 81(42.9) | 60(41.4) | *3:1* | 0.582(0.377-0.901) | **0.015** |
| Hyperlipidemia | No | 181(78.7) | 100(52.9) | 99(67.8) | *2:1* | 0.304(0.199-0.466) | **<0.001** |
|  | Yes | 49(21.3) | 89(47.1) | 47(32.2) | *3:1* | 0.570(0.357-0.912) | **0.019** |
| Heart disease | No | 213(93.0) | 146(77.2) | 124(84.9) | *2:1* | 0.255(0.138-0.470) | **<0.001** |
|  | Yes | 16(7.0) | 43(22.8) | 22(15.1) | *3:1* | 0.423(0.214-0.837) | **0.013** |
| Smoking | No | 160(69.6) | 128(67.4) | 95(65.1) | *2:1* | 0.903(0.597-1.366) | 0.629 |
|  | Yes | 70(30.4) | 62(32.6) | 51(34.9) | *3:1* | 0.815(0.524-1.267) | 0.363 |
| Drinking | No | 188(82.5) | 128(67.7) | 104(72.2) | *2:1* | 0.446(0.283-0.706) | **0.001** |
|  | Yes | 40(17.5) | 61(32.3) | 40(27.8) | *3:1* | 0.553(0.336-0.912) | **0.020** |
| Stroke in relatives | No | 94(63.5) | 130(72.2) | 93(67.4) | *2:1* | 1.494(0.936-2.383) | 0.092 |
|  | Yes | 54(36.5) | 50(27.8) | 45(32.6) | *3:1* | 1.187(0.728-1.935) | 0.491 |
| **Knowledge about infarction** | Less (not at all +no) | 70(30.7) | 86(46.5) | 83(57.6) | *2:1* | 6.348(2.506-16.078) | **<0.001** |
|  | Intermediate | 127(55.7) | 93(50.3) | 51(35.4) | *2:1* | 3.783(1.517-9.439) | **0.004** |
|  | More (more+extremely) | 31(13.6) | 6(3.2) | 10(6.9) | *3:1* | 3.676(1.684-8.023) | **0.001** |
|  |  |  |  |  | *3:1* | 1.245(0.569-2.725) | 0.584 |
| **Personality** | Introverted | 157(71.4) | 71(41.0) | 54(40.0) | *2:1* | 0.279(0.183-0.426) | **<0.001** |
|  | Extroverted | 63(28.6) | 102(59.0) | 81(60.0) | *3:1* | 0.268(0.170-0.420) | **<0.001** |
| **Attitude toward**  **quality of life** | Strongly disagree + disagree | 21(9.3) | 26(13.8) | 10(6.9) | *2:1* | 1.686(0.912-3.118) | **0.001** |
|  | Uncertain | 14(6.2) | 22(11.6) | 23(16.0) | *2:1* | 2.140(1.058-4.328) | **0.034** |
|  | Strongly agree + agree | 192(84.6) | 141(74.6) | 111(77.1) | *3:1* | 0.824(0.374-1.812) | 0.630 |
|  |  |  |  |  | *3:1* | 2.842(1.405-5.747) | **0.004** |

**Abbreviation:** S1= Consent to thrombolysis subgroup; S2= Objection to thrombolysis subgroup 1; S3= Objection to thrombolysis subgroup 2

OR=Odds Ratios

**a 2:1=** Objection to thrombolysis subgroup 1 **:** Consent to thrombolysis subgroup;

**b 3:1=** Objection to thrombolysis subgroup 2 **:** Consent to thrombolysis subgroup;

**Appendix References**

**1. Lipkus IM, Samsa G, Rimer BK. General performance on a numeracy scale among highly educated samples. *Medical decision making : an international journal of the Society for Medical Decision Making*. 2001;21:37-44**

**2. ZY. W. Self-reporting inventory (scl-90). *Shanghai Archives of Psychiatry*. 1984:68-70**
